# StemRegenin 1 Mitigates Radiation-Mediated Hematopoietic Injury by Modulating Radioresponse of Hematopoietic Stem/Progenitor Cells

**DOI:** 10.3390/biomedicines11030824

**Published:** 2023-03-08

**Authors:** You Jung Hwang, Dong-Yeop Shin, Min-Jung Kim, Hyosun Jang, Soyeon Kim, Hyunwon Yang, Won Il Jang, Sunhoo Park, Sehwan Shim, Seung Bum Lee

**Affiliations:** 1 Laboratory of Radiation Exposure & Therapeutics, National Radiation Emergency Medical Center, Korea Institute of Radiological and Medical Sciences, Seoul 01812, Republic of Korea; 2 Center for Medical Innovation of Biomedical Research Institute, Seoul National University Hospital, Seoul 01812, Republic of Korea; 3 Biohealth Convergence, Seoul Women’s University, Seoul 01812, Republic of Korea

**Keywords:** StemRegenin 1, hematopoietic injury, hematopoietic stem/progenitor cells, radiation response, radiomitigation

## Abstract

Hematopoietic injury resulting from the damage of hematopoietic stem/progenitor cells (HSPCs) can be induced by either nuclear accident or radiotherapy. Radiomitigation of HSPCs is critical for the development of medical countermeasure agents. StemRegenin 1 (SR1) modulates the maintenance and function of HSPCs under non-stress conditions. However, the impact of SR1 in radiation-induced hematopoietic injury both in vivo and in vitro remains unknown. In this study, we found that treatment with SR1 after irradiation of C57BL/6 mice significantly mitigates TBI-induced death (80% of SR1-treated mice survival vs. 30% of saline-treated mice survival) with enhanced recovery of peripheral blood cell counts, with the density and cell proliferation of bone marrow components as observed by Hematoxylin and Eosin (H&E) and Ki-67 staining. Interestingly, in vitro analysis of human HSPCs showed that SR1 enhanced the population of human HSPCs (CD34+) under both non-irradiating and irradiating conditions, and reduced radiation-induced DNA damage and apoptosis. Furthermore, SR1 attenuated the radiation-induced expression of a member of the pro-apoptotic BCL-2 family and activity of caspase-3. Overall, these results suggested that SR1 modulates the radioresponse of HSPCs and might provide a potential radiomitigator of hematopoietic injury, which contributes to increase the survival of patients upon irradiation.

## 1. Introduction

Radiotherapy has an important modality for the treatment of cancer patients. Following radiotherapy, radiation can also damage the surrounding healthy tissue, including bone marrow. Furthermore, total body irradiation (TB) causes acute radiation sickness, including hematopoietic, gastrointestinal, and neurovascular syndrome, amounting to a significant threat to public health [1,2,3]. The hematopoietic system is the most sensitive to radiation injury, manifesting as clinical symptoms such as bleeding, infection, and bone marrow (BM) failure [4,5,6]. Radiation causes hematopoietic injury by damaging hematopoietic stem/progenitor cells (HSPCs). Owing to its capacity for self-renewal and differentiation, HSPC transplantation is currently employed to treat hematopoietic injury. However, the limited supply of HSPCs necessitates a strategy for increasing HSPC availability in vitro [7,8,9]. Additionally, agents for recovery and mitigation of HSPC damage are also necessary to increase the survival of patients following irradiation.

StemRegenin 1 (SR1) is an antagonist of the aryl hydrocarbon receptor (AhR) that functions as a cytosolic transcription factor to regulate xenobiotic metabolism and physiological functions such as cell proliferation, apoptosis, differentiation, pluripotency, and stemness [10]. SR1 facilitates the in vitro expansion of human HSPCs and pluripotent-stem-cell-induced generation of CD34(+) hematopoietic progenitor cells and hematolymphoids [11,12,13]. In addition, clinical applications of SR1 for transplantation therapy based on in vitro HSPC expansion have been evaluated [14,15]. Despite the functional activity of SR1 in HSPCs under non-stress conditions being known, its impact on radiation-induced hematopoietic injury remains unclear.

The principal causes of cell injury following irradiation are DNA damage, which is the main aim [16], and apoptosis, which is induced by both extrinsic and intrinsic pathways. The latter is predominantly mediated by the B-cell lymphoma-2 (BCL-2) family [17,18]. Pro-apoptotic BCL-2 family members (PUMA, NOXA, and BAX) cause mitochondrial outer membrane permeabilization (MOMP) to induce apoptosis via caspase-3 cleavage, whereas anti-apoptotic members (BCL-2 and BCL-xL) protect MOMP to inhibit apoptosis by preserving its integrity [19]. The function, maintenance, and operation of the hematopoietic system depend on closely controlled interactions between the pro-apoptotic and anti-apoptotic BCL-2 family members [20]. However, the effect of SR1 on the BCL-2 family in HSPCs following irradiation is poorly understood.

In this study, we examined the impact and action mode of SR1 in modulating HSPCs upon irradiation. These results provide significant insights into the role of SR1 in irradiated HSPCs and may contribute to the development of an effective treatment to mitigate radiation-induced hematopoietic injury.

## 2. Materials and Methods

### 2.1. Animal Experimentation and Radiation Exposure

The C57BL/6 mice (male, 6–8 weeks) were purchased (DooYeol Biotech, Seoul, Republic of Korea), and the Animal Investigation Committee (Korea Institute of Radiological and Medical Sciences, 2022-0043) authorized the animal experimentation procedure. Radiation exposure (1.0 Gy/min) was performed as a single lethal dose of total body irradiation (TBI) using the X-RAD 320 device (Softex, Gyeonggi-do, Republic of Korea).

### 2.2. Radiomitigation by SR1 in Lethally Irradiated Mice

To assess the efficacy of SR1 (APExBIO, Houston, TX, USA), TBI-exposed mice (6 Gy, n = 10/group) were injected at 4 h post-TBI with 100 uL of normal saline or SR1 (30 µg/kg) via intraperitoneal injection three times a week every other day. Survival was monitored for 30 days following irradiation.

### 2.3. Blood Cell Counts in Mice

Mouse blood was collected (200 µL) in EDTA tubes and analyzed with a hematology analyzer, VETSCAN HM5 (ABAXIS, Union City, CA, USA), to acquire neutrophil, lymphocyte, monocyte, white blood cell (WBC), red blood cell (RBC), and platelet counts.

### 2.4. Histologic Analysis of Bone Marrow (BM)

The histologic analysis of BM was performed as reported by Shim et al. (2013). Femurs were fixed with 10% formalin and embedded in paraffin. The sliced tissue sections (4 μm) were stained with Hematoxylin and Eosin (H&E). For analysis of density of BM cells, H&E-stained sections were used to quantify BM cellularity as percent of area without adipocytes per picture. At least three different fields were obtained at 20× magnification using an Olympus microscope and DP-72 digital camera (Olympus, Tokyo, Japan), and analyzed using the I-solution image analysis software (IM Technology, Vancouver, BC, Canada). For immunohistochemistry with anti-Ki-67 (Acris, Hiddenhausen, Germany), following antigen retrieval and blocking of endogenous peroxidase activity (0.3% hydrogen peroxide), sliced tissue sections were sequentially incubated with anti-Ki-67 and anti-rabbit-HRP (Dako, Carpinteria, CA, USA) and developed with diaminobenzidine substrate (DAB kit; Dako, Carpinteria, CA, USA) for peroxidase activity. To quantitatively assess proliferative cells, the number of Ki-67-positive cells per picture was quantitated using I-solution image analysis software version 1 (IM Technology, Vancouver. BC, Canada). Three different fields were investigated with 20× magnification.

### 2.5. Culture and Treatment of HSPCs

HSCPs derived from BM were purchased from ATCC (Manassas, VA, USA) and cultured in StemMACS HSC growth media (Miltenyi Biotech, Auburn, CA, USA). HSCPs treated with SR1 (1 μM) were irradiated at 2Gy using a Gammacell 3000 Elan irradiator (137Cs γ-ray source; MDS Nordion, Ottawa, ON, Canada).

### 2.6. HSPC Population Analysis by FACS

HSPCs were incubated with fluorescence-conjugated antibodies (CD33-PE, CD34-FITC, or CD36-PE) purchased from Biolegend (San Diego, CA, USA) for 30 min on ice. The antibody-treated HSPCs were resuspended in FACS buffer (0.1% BSA and EDTA in PBS) and analyzed using a flow cytometer (BD FACS CANTO II) and the CellQuest program (BD Biosciences, San Jose, CA, USA). A dataset of 10,000 cells was acquired.

### 2.7. Apoptosis Assay

The apoptosis of HSPCs was evaluated using a BD Bioscience detection kit (Waltham, MA, USA). HSPCs resuspended in the binding buffer were stained with annexin V/PI. A flow cytometer (BD Biosciences) was used to examine stained HSPCs after 5 min. Caspase-3 activity in HSPCs was evaluated using a Caspase-3 Assay Kit obtained from Abcam (Cambridge, UK).

### 2.8. Immunocytochemistry (ICC)

HSPCs were fixed with 4% paraformaldehyde and treated with anti-phospho-H2AX (Millipore, MA, USA) or anti-53BP1 (Santa Cruz Biotechnology, CA, USA), which was diluted in blocking buffer (1% BSA/0.1% Triton X-100). Thereafter, either anti-mouse-PE or anti-rabbit-FITC (BD Bioscience) was added as a secondary antibody (1:500). The nuclei were stained with DAPI from Sigma (St. Louis, MO, USA).

### 2.9. Comet Assay

The alkaline comet test kit from Cell Biolabs (San Diego, CA, USA) was utilized to assess DNA damage in HSPCs. The comets were observed with a fluorescence microscope (Olympus IX71, Tokyo, Japan).

### 2.10. RT-PCR

The RNase mini kit obtained from Qiagen (Valencia, CA, USA) was used to prepare the total RNA of HSPCs. The cDNA was extracted from total RNA (1 μg) using RT-PCR in duplicate reactions. Band intensity was evaluated with the Gel DOC image program (Bio-rad, Hercules, CA, USA). The primer sequences and predicted fragment sizes are listed in Table S1 of the Supplementary Materials.

### 2.11. Statistical Analysis

Lethality was assessed using Kaplan–Meier analysis and statistical comparisons between groups were analyzed by Log-rank test. The standard deviation (SD) and standard error (SE) were used to depict the remaining cell and animal data. Two-tailed Student’s *t*-tests were used to determine statistical differences among groups (*p* values < 0.05 were considered significant).

## 3. Results

### 3.1. StemRegenin 1 (SR1) Mitigated Total Body Irradiation (TBI)-Induced Lethality and Pancytopenia in Mice

**Figure 1:**
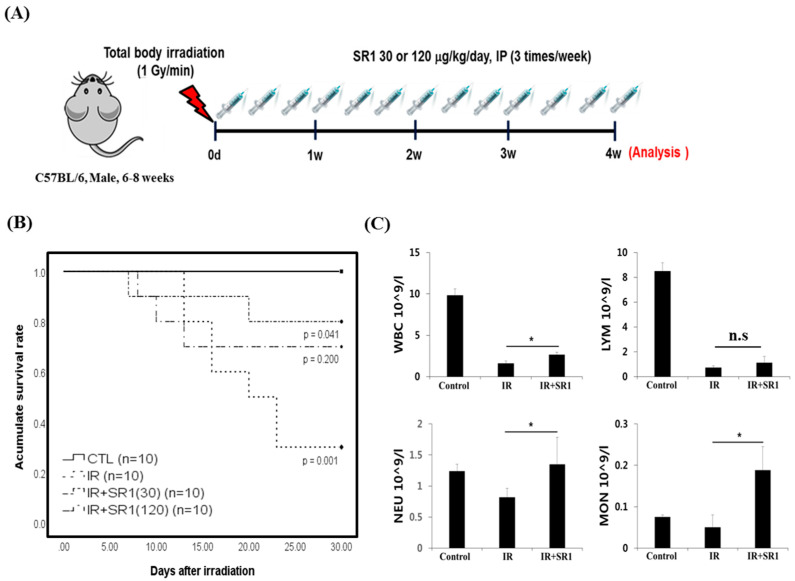
Effect of StemReginin 1 (SR1) on TBI-induced lethality and pancytopenia in mice. (**A**) Schematic of radiation and SR1 treatment for mice. Male C57BL/6 mice (n = 10) were irradiated using a single exposure of 6 Gy of TBI, and either 30 or 120 μg/kg of SR1 was administered by intraperitoneal injection as the indicated treatment (three times per week) after irradiation. (**B**) Kaplan–Meier survival curve depicts the 30-day survival for saline-treated mice (control), irradiated (IR) mice, and mice treated with irradiation with different doses of SR1 (IR+SR1). (**C**) Peripheral leukocyte counts by total differential blood cell counts (WBCs, neutrophils (NEU), lymphocytes (LYM), and monocytes (MOM)) in irradiated mice with or without 30 μg/kg of SR1 treatment. The data are shown as means ± SE (n = 6 per control group, n = 3 per IR group, and n = 6 per IR+SR1 group); * *p* < 0.05 versus irradiated group; n.s: not significant; TBI: total body irradiation.

**Figure 2:**
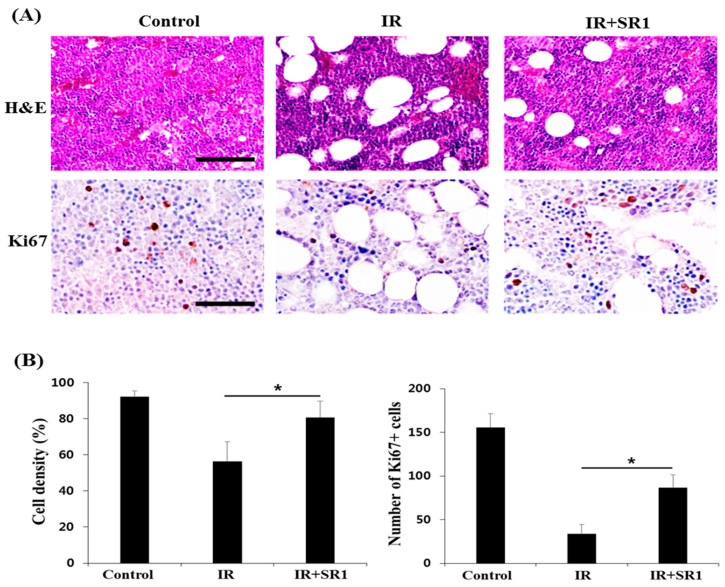
Mitigating effect of SR1 with respect to TBI-induced bone marrow (BM) depletion in vivo. Representative images (**A**) and quantification (**B**) of the effect of SR1 on BM depletion in mice (n = 3 and 8) at 30 days after irradiation. Panels show Hematoxylin and Eosin staining (BM cellularity) and Ki-67 staining (BM cell proliferation) of mouse femurs. Scale bars, 100 μm. * *p* < 0.05 versus IR and IR+SR1 group.

TBI-induced lethality results in the depletion of radio-sensitive hematopoietic elements in BM [9,21]. It is known that SR1, an antagonist of AhR, facilitates the in vitro expansion of human HSPCs for transplantation treatment [11,12]. However, the effect of SR1 in vivo on irradiation-induced lethality is less documented. To investigate whether SR1 affects radiation-induced lethality, mice were treated with saline as the control or SR1 (30 or 120 μg/kg) three times per week after TBI at 6 Gy (LD70/30) (Figure 1A). Kaplan–Meier analysis showed only 30% survival in saline-treated mice after irradiation. In SR1-treated mice after irradiation, we observed statistically significant survival in the 30 μg/kg SR1-treated group (80%), but not in the 120 μg/kg of SR1-treated group (Figure 1B). In addition, with higher concentrations (500 and 1000 μg/kg) of SR1 treatment after irradiation, survival rates were similar to the irradiation-only group. To support the mitigating effect of SR1 in irradiated mice based on the optimized dose (30 μg/kg) of SR1, peripheral blood cell counts representing hematopoietic cells were analyzed. The findings indicated a significant increase in WBC, neutrophil, and monocyte counts but not lymphocyte counts in SR1-treated mice at day 30 compared to those in saline-treated mice (Figure 1C). Histologically, it has been reported that changes in BM cellularity exhibiting massive removal of cellular contents and adipocyte infiltration in bone marrow is a major feature of TBI-induced BM suppression [22]. Therefore, we investigated the effect of SR1 on BM cellular content by histological staining of the femurs after irradiation. As shown in Figure 2A, the irradiation plus SR1 treatment (IR+SR1) group showed a higher density of BM cells with less adipocyte infiltration than in the irradiation-only treatment group. In addition, enhanced cell proliferation was observed in the SR1 treatment group (Figure 2B). Consequently, the data suggested that SR1 mitigated TBI-induced lethality with hematologic recovery following irradiation.

### 3.2. SR1 Treatment Promoted HSPC Expansion and Attenuated the Radiation-Induced Reduction in the HSC Population

**Figure 3:**
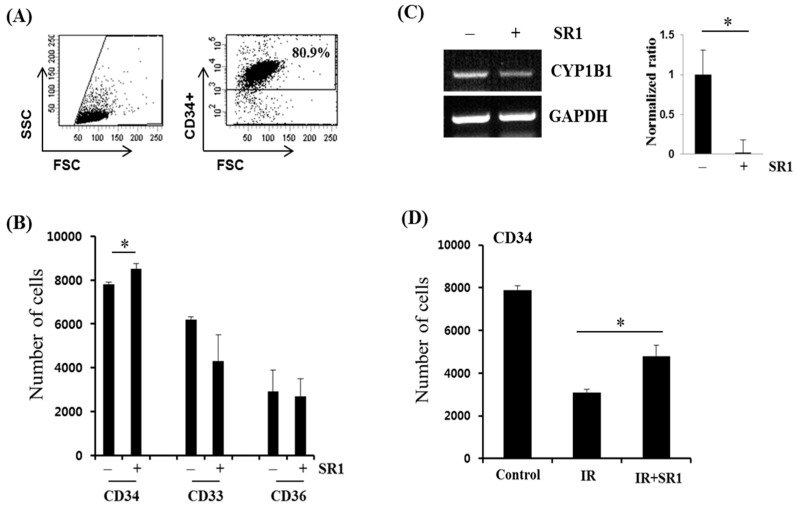
Attenuation of the radiation-induced reduction in HSPC population by Stemneginin 1 (SR1), resulting in HSPC expansion. (**A**) Representative FACS analysis for detecting CD34-positive cells in HSPCs cultured for 5 days. (**B**) Quantification of cells expressing CD34 (HSPCs), CD33 (myeloid), and CD36 (erythrocyte) after SR1 treatment (1 μM) by FACS analysis. A dataset was collected for 10,000 cells. (**C**) RT-PCR analysis of the expression of CYP1B1 (AhR target gene) in HSPCs with or without SR1 treatment. GAPDH was used as an internal control. The data are shown as means ± SDs of triplicate experiments (* *p* < 0.05, two-tailed Student’s *t*-test). (**D**) Quantification of cells expressing CD34 in SR1-treated HSPCs on day 3 after radiation (2 Gy) by FACS analysis. A dataset was collected for 10,000 cells.

As SR1 mitigated TBI-induced BM depletion with increased cell populations, including that of peripheral blood cells for hematologic recovery in vivo, we studied the effect of SR1 on HSPC properties in vitro. To do so, we first confirmed the in vitro expansion of human HSPCs upon SR1 treatment. The concentration of SR1 was based on that in previous studies [11,13]. Flow cytometry (Figure 3A,B) revealed that SR1 enhanced the population of cells positive for CD34, a human HSPC marker [23], but not that of cells positive for CD33, a myeloid marker [24], and cells positive for CD36, an erythrocyte marker [25]. We also observed the increased expression of SCF, an HSPC stemness-related gene [26] In addition, RT-PCR analysis revealed that the SR1-treated group had dramatically reduced expression of cytochrome P450 family 1 subfamily B member 1 (CYP1B1), which is known to be a target gene of AhR [27] (Figure 3C). Interestingly, SR1 treatment enhanced the population of cells expressing CD34 upon radiation (Figure 3D), indicating that SR1 modulated the radioresponse of HSPCs.

### 3.3. SR1 Decreased the Genomic Instability and Apoptosis of HSPCs in Response to Radiation

**Figure 4:**
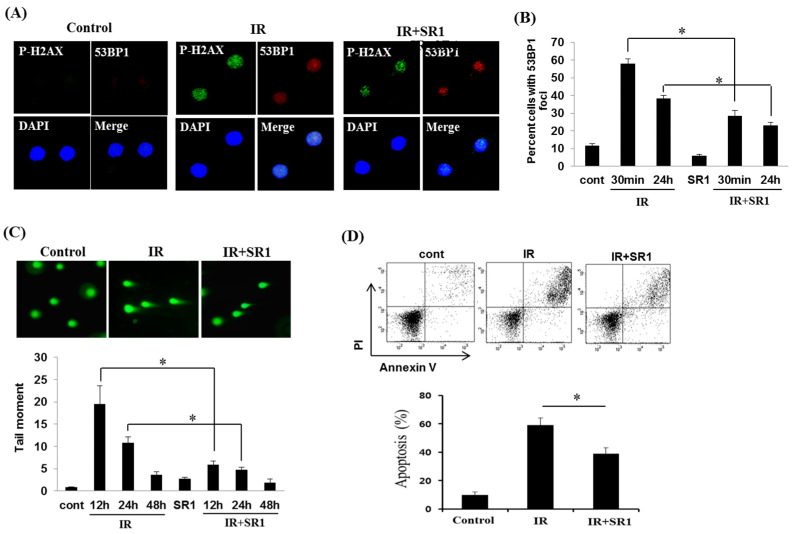
SR1-mediated reduction in genomic instability and apoptosis of HSPCs in response to radiation. (**A**) Immunohistochemical analysis of γH2AX and 53BP1 in HSPCs with or without SR1 on day 1 after irradiation. Scale bars, 50 μm. (**B**) The quantification of percent cells with 53BP1 foci in HSPCs with or without SR1 at indicated times after irradiation. (**C**) Comet assay representing DNA damage (upper panel) and quantification of DNA damage by tail moment in HSPCs with or without SR1 at indicated times after irradiation. (**D**) Number of apoptotic cells on day 3 after irradiation with 2 Gy were measured by annexin V staining using FACS analysis. The data are shown as means ± SDs of triplicate experiments (* *p* < 0.05, two-tailed Student’s *t*-test).

Radiation exposure has significant biological consequences such as cell death and chromosomal instability, resulting from double-strand breaks (DSB) in DNA [18,28]. Immunostaining (Figure 4A,B) revealed an increase in γ-H2AX and 53BP1 phosphorylation, both of which were indicators of DSB [29,30] up to 1 d post-irradiation. Notably, SR1 treatment dramatically attenuated the radiation-induced phosphorylation of both molecules from the initial time point (at 30 min post-irradiation). In support of this result, a comet assay revealed a significant reduction in radiation-induced DNA damage upon SR1 treatment (Figure 4C). Furthermore, apoptosis analysis (annexin V/PI double staining) revealed a decrease in radiation-induced apoptosis in SR1-treated HSPCs (compared to non-treated HSPCs) (Figure 4D), whereas apoptosis was unaffected by SR1 following radiation in other cell types related to HSPC lineage commitment, including Jurkat and ARH77 (Supplementary Figure S1). These results suggest that radiation-induced DNA damage and apoptosis of HSPCs could be selectively regulated by SR1.

### 3.4. Members of the BCL-2 Family Were Involved in SR1-Mediated Alleviation of HSPC Apoptosis Following Irradiation

**Figure 5:**
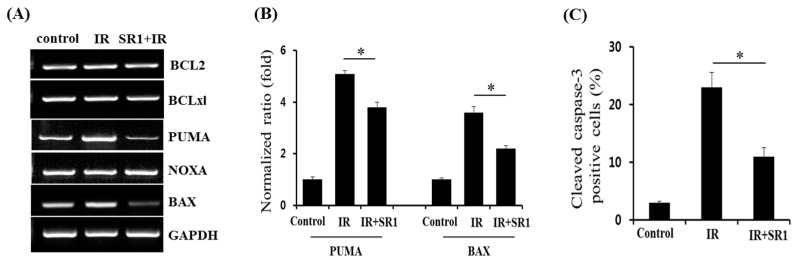
SR1-mediated reduction in the expression of pro-apoptotic BCL-2 family proteins and caspase-3 activity in irradiated HSPCs. (**A**) RT-PCR analysis of the expression of anti-apoptotic BCL-2 family (BCL-2 and BCL-XL) and pro-apoptotic BCL-2 family (PUMA, NOXA, and BAX) members in HSPCs with or without SR1 on day 3 after irradiation. GAPDH was used as an internal control. (**B**) Quantification of PUMA and BAX (pro-apoptotic BCL-2 family) expression. Protein quantification was performed using densitometry. (**C**) Caspase-3 activity assay in HSPCs with or without SR1 on day 3 after irradiation using a cleaved caspase-3 ELISA kit. The data are shown as means ± SDs of triplicate experiments (* *p* < 0.05, two-tailed Student’s *t*-test).

To assess the possible mechanism behind the SR1-mediated inhibition of HSPC apoptosis in response to radiation, we further determined whether SR1 treatment of HSPCs impacts the expression of BCL-2 family proteins, which are known to regulate apoptosis in response to radiation in HSPCs [31,32]. RT-PCR revealed that SR1 dramatically attenuated the radiation-induced expression of PUMA and BAX but not NOXA (pro-apoptotic BCL-2 family members), whereas there was no change in the expression of BCL-2 and BCL-XL (anti-apoptotic BCL-2 family members) in response to either radiation or SR1 treatment (Figure 5A,B). Using the cleaved caspase-3 assay (Figure 5C), we further confirmed that SR1 suppressed the activity of caspase-3, a well-known apoptosis mediator whose activity is dependent on that of the BCL-2 family [17,18,19,20]. Therefore, these results show that SR1 may alleviate the radiation-induced apoptosis of HSPCs via intrinsic apoptosis mediated by pro-apoptotic BCL-2 family members.

## 4. Discussion

Hematopoietic injury due to defects in the self-renewal capacity of HSPCs is the major feature associated with acute radiation sickness following TBI [2,5,6,7,8,9]. Recently, researchers have attempted to treat hematopoietic injury by transplanting HSPCs grown using SR1 [11,15]. In this study, we further supported these studies by demonstrating that SR1 mitigated TBI-induced lethality and pancytopenia, which are associated with reduced genomic instability and apoptosis of HSPCs. We also found that SR1-mediated attenuation of HSPC apoptosis after irradiation was associated with the downregulation of PUMA and BAX (members of the pro-apoptotic BCL-2 family).

SR1 is an antagonist of AhR, a cytosolic transcription factor that participates in the metabolism of xenobiotics, such as environmental toxins and carcinogens, and the regulation of several physiological functions, including cell proliferation, apoptosis, differentiation, pluripotency, and stemness [10]. SR1 facilitates the in vitro expansion of human HSPCs and the pluripotent-stem-cell-induced generation of CD34(+) hematopoietic progenitor cells and hematolymphoid progenitor cells during hematopoiesis [11,12,13]. However, the effect of SR1 on hematopoietic injury has not been investigated. In this study, we demonstrated, for the first time, that SR1 mitigated radiation-induced cell death and improved hematologic recovery, i.e., recovery of WBC, neutrophil, and monocyte counts in a mouse TBI injury model. An increase in the density of BM stromal cells and hematopoietic cells with increased cell proliferation was observed in SR1-treated mice after TBI, further supporting the mitigating effects of SR1. These findings are important, as they demonstrate a radiation countermeasure agent that can be further developed for use against radiation-induced hematopoietic injury. Further studies for translational medicine are required to determine whether these radiation-mitigating effects of SR1 in HSPCs differ between the sexes of C57BL/6 mice, considering that sex can affect the biological consequences of radiation exposure, including biomarker response [33].

The biological consequences of radiation exposure include DNA damage and cell death [18,28]. Radiation generates various DNA-cluster lesions, including DSBs, leading to genomic instability [34]. Concordantly, in irradiated human HSPCs, our data revealed an increase in γ-H2AX and 53BP1 phosphorylation, both of which are known to be DSB markers [29,30], which is reduced by SR1 treatment. To support this, we further confirmed the significant reduction in DNA damage in SR1-treated HSPCs following irradiation using a comet assay; this suggests that SR1 could attenuate the radiation-induced genomic instability. AhR activation by environmental toxins regulates apoptosis of non-hematopoietic cells [35,36]. Indeed, we observed that SR1 increased the population of cells with CD34 expression (human HSPC marker) [23] upon irradiation and suppressed radiation-induced apoptosis of HSPCs after confirming the proper action of SR1 under non-stress conditions, indicated by the increased population of cells positive for CD34 and SCF expression, enhanced HSPC stemness-related gene expression [26], and reduced expression of the CYP1B1 gene [27], as previously reported. In contrast, in lineage-committed HSPCs such as Jurkat and ARH77 cells, the apoptosis ratio was not affected by SR1 after radiation, indicating that SR1 may selectively regulate HSPCs in response to radiation because it is now known that AhR function is cell-type and cellular-context specific [37].

The mechanism underlying radiation-induced HSPC injury, i.e., the derivation of HSPC apoptosis through the p53-Puma-caspase pathway, is well studied [31,32]. A well-balanced interplay between pro-apoptotic and anti-apoptotic BCL-2 family members is necessary for the function and maintenance of the hematopoietic system [20]. The results of these previous studies were corroborated by our findings of PUMA and BAX (members of the pro-apoptotic BCL-2 family) upregulation in irradiated HSPCs, which was dramatically attenuated upon SR1 treatment. In contrast, neither radiation nor SR1 changed the expression of NOXA, which was consistent with a previous report that suggested that NOXA played a modest role in irradiation-induced apoptosis of hematopoietic cells [38,39]. Furthermore, SR1 inhibited the activity of caspase-3, a known apoptosis mediator whose activity is dependent on that of BCL-2 family members [17,18,19], which were also suppressed by SR1. Considering that the intrinsic apoptotic pathway was mostly modulated by the BCL-2 family-dependent caspase-3 pathway via mitochondrial dysfunction, SR1 may alleviate the radiation-induced apoptosis of HSPCs by regulating the intrinsic apoptosis pathway (including PUMA and BAX). Further studies are required to determine a strategy by which AhR-mediated expression of BCL-2 family proteins in HSPC injury can be regulated.

## 5. Conclusions

By analyzing the effects of SR1 on radiation-mediated hematopoietic injury both in vivo and in vitro, we demonstrated, for the first time to our knowledge, that SR1 mitigated BM depletion following irradiation, enhanced hematologic recovery in vivo, and protected human HSPCs from DNA damage and apoptosis induced by irradiation in vitro. These findings contribute to our understanding of the effects of SR1 on HSPCs under stress conditions and could contribute to the development of a radiation-mitigating agent for hematopoietic injury.

## Data Availability

Data are available within the article.

## Supplementary Materials

The following supporting information can be downloaded at: https://www.mdpi.com/article/10.3390/biomedicines11030824/s1, Table S1: Primer sequences used in RT-PCR analysis; Figure S1: Effect of SR1 in radiation-induced apoptosis of Jurkat and ARH77 cells.
