# Supplementary material for: StemRegenin 1 Mitigates Radiation-Mediated Hematopoietic Injury by Modulating Radioresponse of Hematopoietic Stem/Progenitor Cells
Source: Biomedicines. 2023 Mar 8;11(3):824. doi: 10.3390/biomedicines11030824 (PMC10045038; doi:10.3390/biomedicines11030824)
Supplement: Supplementary file 1 [file biomedicines-11-00824-s001.zip › biomedicines-2243255-supplementary.pdf]

Supplementary materials

Hwang YJ *et al.*,

Stemregenin 1 mitigates radiation-mediated hematopoietic injury by modulating radioresponse of hematopoietic stem/progenitor cells

Supporting Information: Table and Figure Legends

Supplemental Table S1. Primer sequences used in RT-PCR analysis

Gene		Primer sequences	Size (bp)
CYP1B1	F	5’-CACTGCCAACACCTCTGTCT-3’	380
	R	5’-CAAGGAGCTCCATGGACTCT-3’	
BCL-2	F	5’-TAGGATTGTGGCCTTCTTTG-3’	170
	R	5;-ACAGTTCCACAAAGGCATCC-3;	
BCL-XL	F	5’-GATCCCCATGGCAGCAGTAA-3’	104
	R	5’-CCCCATCCCGGAAGAGTTCAT-3’	
PUMA	F	5’-GTGTAGAGGAGACAGGAATC-3’	436
	R	5’-GTGTAGAGGAGACAGGAATC-3’	
NOXA	F	5’-AAGAAGGCGCGCAAGAAC-3’	215
	R	5’-CGTGCACCTCCTGAGAAAAC-3’	
BAX	F	5’-TTTGCTTCAGGGTTTCATCC-3’	246
	R	5’-CAGTTGAAGTTGCCGTCAGA-3’	
GAPDH	F	5’-CGAGATCCCTCCAAAATCAA-3’	294
	R	5’-TGTGGTCATGAGTCCTTCCA-3’	

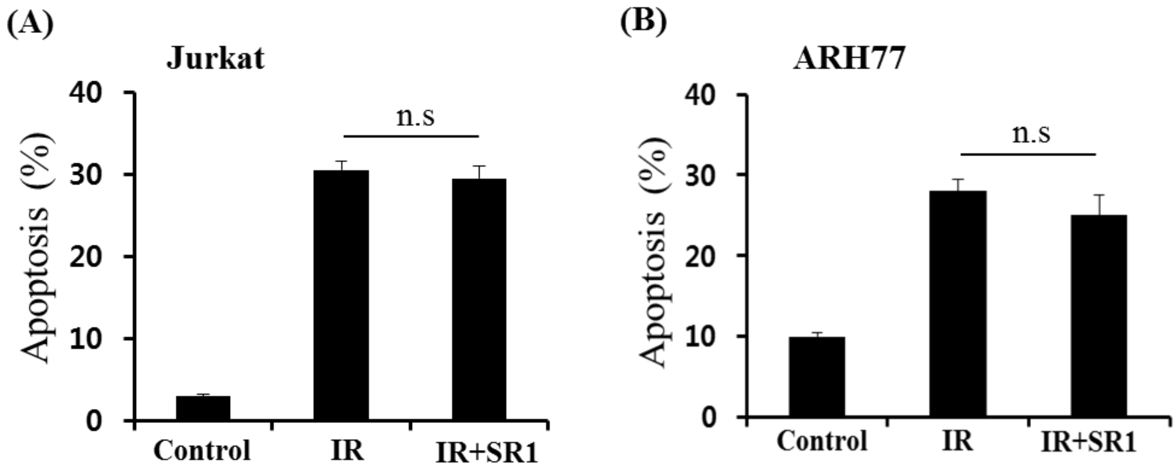

Supplemental Figure S1. Effect of SR1 in radiation-induced apoptosis of jurkat and ARH77 cells. Apoptotic cells of jurkat (A) and ARH77 (B) on day 3 after irradiation with/without SR1 were measured by annexin V staining using FACS analysis. The data are shown as means ± SDs of triplicate experiments (*$P < 0.05$, two-tailed Student's t-tests).
